# Bromide of Potassium and Quinia Combined, in the Treatment of Intermittent Fever

**Published:** 1875-11

**Authors:** T. S. Dekle

**Affiliations:** Thomas County, Georgia


### BROMIDE OF POTASSIUM AND QUINIA COMBINED, IN THE TREATMENT OF INTERMITTENT FEVER.

By T. S. DEKLE, M.D., of Thomas County, Georgia.

*Read before the South Georgia Medical Society, at Bainbridge, Ga., Sept. 26, 1875.*

Heretofore quinia was considered the specific in the treatment of intermittent fever and other malarious manifestations, combined, however, at times with other remedies to meet symptomatic expressions of individual cases, but it was considered the Sampson in treating the disease itself. That it is indispensable to meet the cause of the disease, I do not propose to deny; but that its virtues are adequate to the cure of the disease, I claim is not founded upon its medicinal virtues as directed to the pathological condition of intermittent fever. I am aware that this will be considered a very bold and presumptuous proposition; but I appeal to the experience and judgment of those very ones that perchance might treat this proposition lightly. How many cases have you that did not readily yield to the treatment of quinia? or have you never been disappointed? I commenced the use of quinia in intermittent and other malarious diseases as confident of its power to control the paroxysms as the most sanguine supporters of its virtues, and yet often was chagrined to know that, regardless of my efforts, the paroxysms would return either at their regular period, or in a few days thereafter, surely and steadily subverting and sapping the foundation of the patient's vitality and strength, paving the way to more serious and complicated sequels, either to result in a miserable existence or consign him to an untimely grave.

Quinia is claimed to be antiperiodic; but can our experience substantiate its antiperiodic properties? If so, upon what ground do we attribute its antiperiodic virtues? Intermittent fever is supposed to be due to poisonous emanations from low, marshy localities, which are absorbed into the system, and which produce that abnormal condition known as intermittent fever. Now, as the causation of this disease is due to malaria, or, as is said (in Flint's practice of medicine, by Professor Salisburg,) to be due to "introduction into the system of cells of spores emanating from certain species of algoid plants, called *palmellas*, which belong to the lowest known vegetable organisms." To these species of plants he applies the generic name "*gemiasma*." Now, as the presence of these *cells* or *miasm* within the system produces the paroxysm, and the paroxysm manifests certain symptoms indicating an irritation of the *nervous centres*, producing spasm of peripheral vessels, or merely manifesting it by general aches and pains, which is followed by fever, it follows that the first rational indication in the treament is to remove the cause of these abnormal phenomena; or, in other words, to administer an antidote to the poison. Clinical, scientific, and experimental knowledge suggest quinia as the antidote; but does clinical, scientific, and experimental knowledge establish the fact that, to remove the cause, cures the effect it has produced? If so, the sum and substance of the province of the physician would be to administer specific antidotes for specific poisons.

But from the great body of medical truths sparkles the fact that the removal of the cause does not remove the effect it has produced, although the removal of the cause is essential in order to treat the effect. An objection to this may be offered by claiming the fact that the removal of the cause very often results in a cure, and if the removal of the cause does not cure the effect, wherefore the cure in numerous cases by simply removing the cause? The answer is found in the great medical fact, in the "*vis medicatrix naturæ;*" or, in other words, the recuperative powers of the system are not sufficiently compromised as to disable its power to cure itself. But to the question, is quinia an antiperiodic positively? In the beginning of the use of Peruvian bark, the method adopted for its use was to administer a full dose immediately before the paroxysm, "not with the hope of preventing" the fit, "but in order to operate more certainly upon the succeeding" one. (Stille—Therapeutic and Materia Medica.) The present plan of administering the remedy by authors is to administer it "as near as possible to the paroxysm which has passed" (Flint.) Stille says, "that the anti-febrile influence of quinia does not coincide with its physiological operation, either in time or in degree." A dose of six grains, for example, is sufficient to prevent the occurrence of a febrile paroxysm fifteen hours afterwards, and yet its direct and sensible action upon the system may be imperceptible, or, if decided, may entirely have passed away before the period of the next paroxysm. This fact, which modern observation and experiment have rendered positive, refutes completely the notion that the antiperiodic operation of bark is explicable by its sedative influence upon the nervous system, and leaves us again without other resources than to adopt the hypothesis that quinia eliminates a morbid poison from the system, or to take refuge under the convenient cloak of ignorance, "the recognition of a specific virtue, *virtutem febrifugam* in the bark." Intermittent fever is a periodical paroxysm, caused by an agent that acts on the nervous system, producing a spasmodic action of the capillary system, and very often the muscular system. The mutations of periodical phenomena are perceptible from the stupendous throes of nature's mightiest display to the simplest manifestation in animate or inanimate things. No one denies but there is a cause to every such phenomena. Consider the category of diseases, from the most serious to the simplest manifestations, and is there not a feature of periodicity about all? Yea, even in the most imaginary condition of health periodical expressions are prominent. Will not the experience of every one coincide, that all spasmodic diseases are periodic? From the known physiological action of quinia, would it be rational to administer it in spasmodic affection in order to arrest its periodical expression? No such virtues are claimed for it; and yet it is given in intermittent fever with a view to arrest a periodical paroxysm which is spasmodic. But says one, the administration of quinia very often cures the paroxysm. True, but does that prove as a consequence that it is directly antiperiodic? or would it not be more rational to attribute its antiperiodic virtue to its power over the cause, and not over the effect, which a *priori* would negatively result in a cure?

Therefore we conclude that quinine is not possessed of virtues, the physiological action of which would positively arrest a spasmodic paroxysm, but the result of its action upon the excitant cause neutralizing it, would, by this action, leave the system free from the cause of a periodical excitant, thereby negatively becoming antiperiodic. Can we not conceive of an agent, the action of which would produce a certain effect, and were that agent removed the effect would be in proportion to the power of the agent, and the resistance of the object? And can we not further perceive that were that agent again to react, that the resisting power of the object would be weakened, and what first would hardly be appreciable would, by repeated attacks, produce an effect, which of itself would become a secondary cause, producing phenomena analagous to the first? Suppose a first cause produce a lesion, then remove the cause, would not the lesion itself express the pain that was produced by the first cause? Then suppose the medicinal and chemical action of a remedy which would be indicated in the first cause, although the effects of the primary cause expressed the symptoms, would it be rational to administer the remedy for the effects when the physiological action of that remedy contra-indicated its use? Miasma does not alone produce a chill and then a fever, nor does it alone produce periodical and spasmodic attacks; and it is well known that quinine does not arrest all periodical attacks, and it is equally as well established that the system gets habituated to certain periodicities having once experienced a paroxysm, predisposes to another. With these facts looming up as pointers to direct our mind in inquiring, with the known physiological action of quinine, is it not rashness to disregard the silent though ominous mutterings of those noble heroes of science, who have handed these facts down for our benefit. Now the question arises if quinine is directed alone to the poisonous or malarious agent that causes intermittet fever, what agent is directed to meet the effect of that poison, or, in other words, to meet the physiological manifestation of the disease? Bromide of potassium is a nervo-sanguine sedative, promotes sleep, prevents convulsions and spasms, palliates pain, and counteracts the element hyperæmia, has a sedative action on the excito-motor function of the spinal marrow. Does not the known pathological condition of intermittent fever indicate this remedy to meet the effects of the morbific agent? If true, and the hypothetical virtues of quinine are also true, is not the combination incomparably more rationally indicated than quinine alone? Quinine anti-malaric, naturalizing the cause. Bromide of potassium anti-spasmodic, and by its direct sedative action, positively anti-periodic, controlling the effect of the disease. These we conclude, by virtue of their combined effect, to be more rationally indicated than either alone.

I now submit to your consideration some cases in which I have used the remedy:

Case I.—November 9, 1874.—S., colored girl, aged eight. Called in; found her in a comatose condition, moaning, spasmodic manifestations at intervals of about ten minutes, pulse flickering, thready and one hundred and twenty, pupils of the eyes dilated, with globe turned upward, extremities cold, a clammy perspiration over the forehead. History—was informed that she had been having chills and fever during the fall; had not had any medical aid prior; had used usual remedies for chills without averting them; did not have any uneasiness about the case until this morning; she was playing about as usual, and had gotten off some distance from the house, when she was heard to utter a scream as if frightened; found in convulsion, lying on the ground. Ascertaining that her bowels were bound, I immediately administered a dose of calomel, and in a few minutes a dose of bromide of potassium, using the usual method in chill, heat, counter irritation, etc.; left quinine to be commenced in two hours.

10th Nov.—Was amazed to find her still in the semi-comatose condition, having at no time rallied; having slight spasm; slept none during the night; kept up the moaning first spoken of. Was told that on account of her inability to swallow, they had given but little medicine since my visit; calomel had acted. Pouring out some solution of potassium, attempted to administer it, but found when the fluid was poured in the mouth, she made no attempt to swallow, and came very near strangulating. I considered the case hopeless, but in the dilemma, after finding that the reflex action of the fauces was partially paralyzed, considering the unusual length of time she had been in that condition, attempting to arrive at some plan of treatment, the idea first occurred to me to use the combined effect of bromide and quinine per enema. I commenced the treatment forthwith, by doubling the quantity used in each per orem, in a little starch water. In about an hour her pulse began to grow fuller and softer, the extremities to warm, and in the course of two hours she was dozing quietly. The change brought about so suddenly, inspired me to continue the use of the remedy. Left with the instructions to carry out the prescription every four hours.

11th.—Pleased to find her rational; had taken some nourishment; my prescription had been carried out; had no symptom of spasm after commencing the remedy. This being the regular chill day, I continued the use of bromide and quinine as commenced. Left, requesting to be informed immediately if she had a chill; if not to come the following morning to my office to let me know her condition.

12th.—Reported no chill, no fever; lively but weak; continued the combination—the combination in solution; cured.

Case II.—December 3, 1874.—W. S.. white, aged three years. Quotidia intermittent fever; had been affected several days; convulsion in chill; had taken quinine, etc. I gave him:

| | |
|---|---|
| ℞.—Quinine, . . . . . . . . | gr. 24 |
| Elix. vitt., . . . . . . . | gtt. 24 |
| Water, . . . . . . . | fl. oz. 3 |
| Mix, then add Bromide of Potassium, . | gr. 72 |

Sig. Teaspoonful every three hours until he has taken four doses during the day, to repeat the same way each day, until the paroxysms are nipped, then dose morning and night.

Had no more chills after commencing the treatment; cured.

Case III.—May 16th, 1875.—H. C., white, aged two years.

Had been having chills for about nine months; had taken quinine, and numerous other remedies; called in the first time; gave him:

| | |
|---|---|
| ℞.—Quinine, . . . . . . . . | gr. 24 |
| Elix. vitt., . . . . . . . | gtt. 24 |
| Water, . . . . . . . | fl. oz. 4 |
| Mix, then add Bromide of Potassium, . | gr. 96 |

Sig. Teaspoonful four times a day until he missed the paroxysm, then twice a day for some time after.

Had no other chill after commencing treatment; cured.

Case IV.—June 8, 1875.—B., mulatto infant, fifteen months old. Had had two chills:

| | |
|---|---|
| ℞.—Quinine, . . . . . . | gr. 12 |
| Elix. vitt., . . . . . . . . | gr. 12 |
| Mix, . . . . . . . . | fl. gtt. 2 |
| Mix, then add Bromide of Potassium, . | gr. 48 |

Sig. Teaspoonful four times.

No more chills; cured.

Case. V.—June 8, 1875.—F., white infant, aged eighteen months. Had been sick with cholera morbus and diarrhœa; was taken with chills and fever three days before; had taken quinine, calomel, etc.

| | |
|---|---|
| ℞.—Quinine, . . . . . . . | gr. 12 |
| Elix. vitt., . . . . . . . | gr. 12 |
| Water, . . . . . . . | fl. oz. 2 |
| Mix, then add Bromide of Potassium, . | gr. 48 |

Sig. Teaspoonful four times a day. Promptly cured.

Case VI.—June 20, 1875.—R. P., negro woman, aged seventy years. Reported to have been sick with intermittent fever for some time; had used domestic remedies to no avail. Gave her:

| | |
|---|---|
| ℞.—Quinine, . . . . . . . | gr. 20 |
| Elix. vit., . . . . . . . | gr. 20 |
| Water, . . . . . . . | fl. oz. 4 |
| Mix, then add Bromide of Potassium, . | gr. 110 |

Dose. Tablespoonful four times a day.

Had no more fever after commencing remedy; cured.

Case VII.—June 21, 1875.—L. R., mulatto woman, aged twenty-two years. Reported at office; stated she had been having severe chills and high fever, for about a week, every other day. Was first taken, prior to chills, by suppression of menses; attributed the suppression to having bathed her feet in cold water whilst very warm; had been laboring during the day. After giving her some remedies for her menstrual trouble, I gave her the combination of bromide and quinine. Had no more chills; cured.

Case VIII.—June 22, 1875.—B. W., mulatto man, aged sixty years. We called to see him, being confined to his bed by double tertian fever. During fits, delirious; had been sick about ten days; had taken various remedies with no benefit. Treatment besides the combination, gave remedies to regulate bowels, as they were inclined to run off. Had no more chills after commencing treatment; cured.

Case IX.—June 28, 1875.—B., colored child, aged two years. Father reported at office for prescription; stated she had been sick about ten days; was attacked first with diarrhœa, subsequently with chills and diarrhœa. For diarrhœa gave:

| | |
|---|---|
| ℞.—Calomel, . . . . . . . . . | gr. 3 |
| Ipecac, pulv. . . . . . . . | gr. 4 |
| Ext. Hyosciam. . . . . . . | gr. 4 |
| Prep. Chalk, . . . | gr. 30—M. ft. ch. no. |

Twelve—one every three hours.

For chills, I gave:

| | |
|---|---|
| ℞.—Quinine, . . . . . . . . . | gr. 12 |
| Elix. vit., . . . . . . . . | gr. 12 |
| Water, . . . . . . . . | fl. oz. 2 |
| Mix, then add Bromide of Potassium, . | gr. 64 |

Sig. Teaspoonful four times a day, until paroxysm is missed, then teaspoonful twice a day.

No more chills after commencing treatment; cured.

Cases X and XI.—June 30, 1875.—C. B., colored woman, aged twenty-two years, and — W., mulatto child, aged two years. Both cases had been having chills for several days, the child going into convulsions during the paroxysms. Gave the combination, with the prompt results of the cases preceeding them, having no more chills after commencing the remedy; cured.

Case XII.—July 4, 1875.—L., mulatto boy, aged eighteen months. Called to see him; had been having very high fever for several days; had no chill that its parents could discover. Gave the prescription as usual. Cured promptly.

Case XIII.—July 4, 1875.—C. B., colored man, aged fifty years. Was sent for to see him by his employer; had been having chills for some time; had so affected him as to confine him to his bed; could not walk about without fainting; had taken calomel and quinine; found him in the decline of the febrile stage. Gave the combination, with the same results of other cases, that of arresting the paroxysms promptly.

CASES XIV AND XV.—July 20, 1874.—W. J. and F. B., aged respectfully fifty-five and fifty years, mulatto and black. Both cases had been having chills for several days. Gave the combination to each, with the results of arresting the paroxysms promptly without a return.

CASE XVI.—July 27, 1875.—C. W., colored man, aged twenty-five years. Had been having intermittent fever for about a week—severe tertian form:

| | |
|---|---|
| ℞·—Quinine, . . . . . . . | gr. 40 |
| Elix. vit., . . . . . . . . | gr. 40 |
| Water, . . . . . . . | fl. oz. 10 |
| Mix, then add Bromide of Potassium, | gr. 400 |

Sig. Tablespoonful four times daily until the paroxysm is missed, then twice a day.

Had no attack after commencing the remedy, and without precaution went about his business the day for paroxysm, without feeling any inconvenience; cured.

CASE XVII.—July 27.—W. E., girl aged eight years. Had been having double tertian fever about a week; sent for and found her, on first visit, in a semi-comatose delirious condition; fever very high; gave her a calomel and rheubarb purgative immediately, and gave the combination as in other cases. Recovered without another attack.

I have administered the remedy in about sixty cases, with the same results, viz: promptly arresting the paroxysm; and those to whom I have administered the combination have become so much pleased with the result, that they have begun to come to me for it to give in their families, stating that they never have had any thing to arrest a chill and fever so promptly.

